# The hidden costs of installing xpert machines in a tuberculosis high-burden country: experiences from Nigeria

**DOI:** 10.11604/pamj.2014.18.277.3906

**Published:** 2014-08-05

**Authors:** Saddiq Tsimiri Abdurrahman, Nnamdi Emenyonu, Olusegun Joshua Obasanya, Lovett Lawson, Russell Dacombe, Muhammad Muhammad, Olanrewaju Oladimeji, Luis Eduardo Cuevas

**Affiliations:** 1 Tuberculosis and Leprosy Control Programme Unit, Department of Public Health and Human Services, Federal Capital Territory, Abuja, Nigeria; 2 Zankli Medical Centre, Abuja, Nigeria; 3 National TB, Leprosy & Buruli Ulcer Control Programme, Nigeria; 4 Liverpool School of Tropical Medicine, United Kingdom

**Keywords:** Tuberculosis, Xpert-Installation, low resource –settings, hidden cost, operational research

## Abstract

**Introduction:**

Since the endorsement of GeneXpert MTB/RIF by the WHO, many countries have embarked on implementing this technology. *Objective:* We outline the cost of installing GeneXpert in district hospitals in Abuja, Nigeria.

**Methods:**

We prospectively documented costs related to the installation of GeneXpert at five sites. Costs were collected from receipts received from suppliers and normalized to USD 2012 values.

**Results:**

Costs were often identified after initiating installation for many reasons. Installation varied widely between sites with sufficient space and power supply; sites with insufficient space or power supply and costs not directly associated with site installation. The basic cost for installation was USD 2,621.98 per machine. Sites that required additional space cost close to USD 7,000.00.

**Conclusion:**

Space and power requirements have a significant effect on installation costs. Countries need to carefully consider the placement of Xpert machines based on the quality and size of the available infrastructure.

## Introduction

Since the World Health Organization WHO endorsement of Xpert MTB/RIF in December 2010 and its subsequent recommendation to health authorities to roll-out the technology in phases within the context of national plans, many high Tuberculosis (TB) burden countries have embarked on implementing the technology to improve the management of TB [1]. Xpert allows a rapid diagnosis of TB and a presumptive diagnosis of multi drug resistant TB (MDR TB) without requiring the comparatively sophisticated laboratory facilities and complex methodologies of other established tests such as culture and line probe assays.

In addition to the capital equipment expenditure and recurrent costs for the cartridges and calibrating machines [2], there are costs associated with upgrading laboratories to receive the new Xpert MTB/RIF machines. These costs are likely to be inversely proportional to the strength of the laboratory infrastructure and to vary across locations [3]. This information however is critical when an institution is planning the introduction of Xpert, and an estimate of the likely costs will inform scale-up decisions. While there are large numbers of studies on the accuracy of Xpert, there is no literature describing the costs associated with its installation in resource -limited settings [4].

WHO in its 2012 global TB report ranked Nigeria as the country with the 10th highest burden of TB with characteristics of a low resource setting [5]. The Federal Capital Territory (FCT) TB and Leprosy Control Programme (TBLCP) was established in 1992 and currently provides 39 treatment and 22 diagnostic centers. In 2012 it diagnosed 2111 patients but, despite a rapid expansion since inception, the case detection rate is still low (at 58%) and below the Nigerian target of 70%. In 2010, the FCT TBLCP purchased 5 new Xpert machines as part of a programme to increase case detection. The study presented the opportunity to describe the costs associated with the introduction of Xpert in selected district hospitals within this context.

## Methods

Five four-module Xpert instruments were received by the FCT-TBLCP in January 2012 and installed from April to July 2012 in district hospitals of the Local Area Councils (LAC) of the FCT. The equipment was part of a TB-REACH-funded project (STOP TB Partnership, Geneva) to test a new approach to increase TB case detection amongst adults in the FCT and the hospital selected had to be located outside Abuja's metropolitan area. The equipment was purchased at the Foundation for Innovative New Diagnostic (FIND) price of 17,500 USD per instrument, computer and freight on board. The project developed a network of community health extension workers who conducted house-to-house visits to identify adults with symptoms of TB and motorbike riders who linked the teams to the laboratory facilities by transporting specimens. Patients were tested using smear-microscopy and smear-negative patients were further tested with Xpert using an interim diagnostic algorithm. The management of the patients was conducted by the programme using its treatment algorithms [6].

During its planning phase, the project assessed the LAC hospital laboratories that routinely conducted smear microscopy to select those considered to have suitable facilities for the introduction of Xpert. Assessments were conducted by a team of laboratory staff, doctors and programme managers working with the Federal Ministry of Health, the FCT TBLCP and Zankli Medical Centre, a private TB research laboratory which supports the NTBLCP and acts as a reference diagnostic centre for the programme. The assessments documented the availability of space to accommodate the instrument and accessories, including a portable computer and bar reading stand, bench space, a backup system for electricity and the security of the premises. Laboratories also had to have at least two laboratory staff were willing to participate who had basic computer literacy for data entry, processing and printing of results.

The costs incurred from the installation were prospectively documented. Costs were documented from receipts received from registered suppliers and normalized to United States Dollar (USD) 2012 values by using the mean exchange rate across the year (USD 1 = 156 Nigerian Naira). Costs were stratified in three categories: - Cost incurred to install the machines for laboratories with sufficient space and adequate electricity supply. These costs were considered the basic expense required for installation and were the same for all five laboratories. - Costs not directly associated with site infrastructure, including custom clearance and training. - Costs incurred into sites subsequently considered having inadequate electricity supply or insufficient space to accommodate additional equipment needs identified during the installation. Recurrent costs for running and maintaining the instruments are not included (e.g. maintenance and fuel for generators and module calibrations).

**Ethics**: This study was approved by the Health Research Ethics Committee of Federal Capital Territory, Abuja-Nigeria and Liverpool School of Tropical Medicine United Kingdom.

## Results

A wide range of costs were incurred during the installation of the instruments. Some of these problems were not foreseen before initiating the project and costs often snowballed due to variety of reasons. For example, despite the survey, it was soon recognized that the electricity backup system provided by the hospitals still allowed short electricity interruptions before a generator re-instated the supply. In turn, the extra equipment needed to secure uninterrupted electrical supply required additional laboratory space.

The main basic costs incurred to install the machines in the laboratories are described in Table 1. These included minimal upgrade of the laboratory premises, painting and reinforcement of working benches, minimal plumbing, additional chairs, extension cables and sockets, voltage stabilizers, inverters and truck batteries, a printer and a refrigerator. Computers required antivirus software and equipment was engraved and secured with cables to prevent thefts. Additional bench space for the equipment and processing of specimens was required by staff in all sites. Custom clearance required payment of a handler despite being duty free and transport to the facilities. The total of these costs amounted to USD 2621.98 per laboratory.

**Table 1:** Cost of installation of Xpert in laboratories with sufficient space

	Cost (USD)
Custom clearance	25.64
Working bench	102.56
Chairs	51.28
Stools	96.15
Refrigerators	141.03
Extension cables	19.23
Printer cable	6.41
Printer	160.26
Tables	19.23
Engraving of equipment	19.23
Anti-virus	25.64
Air-conditioners	192.31
Voltage stabilizer	96.15
Inverters/Batteries and accessories	1,666.86
**Total**	**2621.98**

In two laboratories we purchased generators because the hospitals’ own generators were broken. The electrical equipment (invertors, batteries etc) was bulky and required more space than anticipated and was not possible to accommodate them within the laboratory premises. It became evident that the added equipment overwhelmed the laboratory and could not be accommodated into the existing premises.

We therefore purchased and furnished cargo containers to provide additional bench, storage and office space. Refurbishment included air conditioners, as temperatures are usually >30°C. The costs for the sites requiring additional premises are shown in Table 2. These additional costs ranged from $2,621.98 to $9,716.21.

**Table 2:** Additional costs of installation of Xpert in laboratories without sufficient space or electricity supply

	Cost (USD) by Laboratory		
	Lab 1	Lab 2	Lab 3
**Container**	4,743.59	4,743.59	
Slabs [to support the container]	102.56	153.85	
Sun shield	-	224.36	
Transportation of container	512.82	173.08	
Floor fittings	254.49	237.18	
Carpentry	660.90	557.69	
Electricity	327.88	368.59	
Plumbing	299.68	224.36	
Generator	192.31	-	192.31
**Total**	**7,094.23**	**7,126.28**	**192.31**

## Discussion

The WHO technical and operational guidance on Xpert [2] provides annual itemized budget calculations [4] based on the instrument selected. District hospitals in Nigeria however have multi-purpose diagnostic laboratories where space is at a premium and have limited resilience to adapt to receive new technologies. The guidance costs of installing the Xpert instrument differs from the costs we experienced in this project. For example, the costs of securing an uninterruptable supply of power were three times higher (USD 1666.86) than estimated in the guidance (USD 500). Although services are supposed to have a minimal of equipment, this is often in poor condition. Despite the laboratories indicating they had the minimum equipment needed, we had to purchase capital items such as refrigerators, air conditioners and in two cases generators to allow Xpert services to function. Even without generators the costs of installing the machines was USD 1,921.99 per site, which is considerably more expensive that the guidance estimates. Conversely, the cost of training was lower in our case (USD 2,605.19), than the guidance estimate of USD 5,000, which was due to the availability of in-house expertise for installation and training.

Finding the space required for the machines was a common problem, with the FCT health and human services having no space in two sites to build or expand the existing laboratories and competing priorities from other projects and programmes. Consequently, with the need to implement the project as quickly as possible, the procurement of cargo containers was the only option available to us. This added, on average, an additional USD 6,792.31 per site. Studies looking at the cost effectiveness of Xpert in India, South Africa and Uganda reported variations in the infrastructure cost [3, 7, 8]. Although the studies did not detail the reasons for these differences, they are likely to reflect the national laboratory infrastructure and the level of the health system where they are placed (tertiary or secondary).

## Conclusion

Our findings in Abuja demonstrate that countries need to carefully consider the placement of Xpert machines based on the quality and size of the laboratory infrastructure to ensure that additional placement costs are controlled. This echoes the concerns raised at the 2012 fourth Global Laboratory Initiative partners meeting, which observed that, although users were pleased with Xpert MTB/ RIF, there were concerns about the price of the technology; especially in terms of accelerated and sustained roll-out in both low- and middle-income countries.
